# Physiology of the Circulation

**Published:** 1860-02

**Authors:** 


### ARTICLE 3.

### PHYSIOLOGY OF THE CIRCULATION.

A recent lecture, by John C. Dalton, Jr., M. D., on the above topic, published in the N. Y. Monthly Review, presents several points worthy a reprint.

Among others are the effects of a stoppage of respiration on the circulation—especially through the arterial system and cardiac cavities. As is usually described, the disturbance which results is principally because the blood finds a difficulty in passing through the lungs, and so distends the right auricle and great veins, and finally presses back on the capillary system. Experiments of Dr. Dalton show that it is not the great veins that become distended after the stoppage of respiration, but that the arterial system first becomes distended then the aorta, then the left cavities of the heart.

By artificial respiration kept up on a dog poisoned with woorara, Dr. Dalton observes that: "when the lungs are allowed to collapse unærated blue blood passes through the lungs, so that the blood in the left auricle is completely dark colored in thirty seconds"—that, "there is a slight swelling of the right and left cardiac cavities, soon after suspension of respiration"—that, "the pulmonary artery and aorta are also distended at this stage," and that, "there is no sensible distension of the vena cava." The unærated blood passes through the auricle and ventricle, the aorta and arterial system to the capillaries which "will not allow it to pass."—Stagnating in the capillary system "the blood begins to exert a backward pressure on the arteries. From the arterial system the engorgement extends to the aorta, and from the aorta to the left cavities of the heart. This state of things continues as long as the action of the ventricle continues sufficiently vigorous to give the blood an impulse towards and into the arteries. But the continued distension the heart suffers, soon begins to paralyze its action more effectually, and at the same time it can be seen that the engorgement of the arterial system diminishes, while that of the heart increases. The regular rythmical action of the left ventricle stops, it no longer discharges any blood into the aorta; and the aorta and arteries generally, by the elastic resiliency of their own walls, and the slow passage of the venous blood through the capillaries, gradually relieve themselves of the load which they contained, and return to their original size."

The effect of post mortem contraction of the cardiac fibres is to obliterate the cavity of the left ventricle and empty it of its blood. The blood is forced into the venous system and into the right side of the heart. This is the appearance a few hours after death.

---

Powder Marks.—Prof. Busch, of Rome, recommends as a local application to remove grains of powder imbedded in the skin after powder wounds, a solution of Corrosive Sublimate gr. v. in aq. ℥ i. On the removal of the scabs after pustulation by this agent, the grains come with it, and the subjacent surface is left smooth and healthy.

